# Supplementary material for: Model predictive game control for personalized and targeted interactive assistance
Source: Commun Eng. 2026 Feb 24;5:57. doi: 10.1038/s44172-026-00605-8 (PMC13013598; doi:10.1038/s44172-026-00605-8)
Supplement: Supplementary file 2 — Supplementary File Model predictive game control for personalized and targeted interactive assistance [file 44172_2026_605_MOESM2_ESM.pdf]

Supplementary File
Model predictive game control for personalized and targeted interactive assistance

Abdelwaheb Hafs, Anaïs Farr, Dorian Verdel,
Olivier Bruneau, Etienne Burdet and Bastien Berret

Supplementary Note 1

In this supplementary note, we present a general dyadic affine-quadratic case that can be used to model more complex interaction dynamics. Specifically, when the actual states and inputs of the robot and human systems are modeled along with their physical connection (e.g., as spring-damper elements), the inputs from the robot and the human will influence different states of the coupled human-robot system.

As in the main text, the control $\mathbf{u}(t)$ combines the inputs from the human agent h and robot agent r. More explicitly, we assume that $\mathbf{u}(t)$ can be written as:

$$\mathbf{u}(t) = \mathbf{S}_{\mathrm{r}}\mathbf{u}_{\mathrm{r}}(t) + \mathbf{S}_{\mathrm{h}}\mathbf{u}_{\mathrm{h}}(t)\,, \tag{1}$$

where $\mathbf{S}_{\mathrm{r}} \in \mathbb{R}^{m\times m_{\mathrm{r}}}$ and $\mathbf{S}_{\mathrm{h}} \in \mathbb{R}^{m\times m_{\mathrm{h}}}$ are reshaping matrices, with $\mathbf{u}_{\mathrm{r}}(t) \in \mathbb{R}^{m_{\mathrm{r}}}$ and $\mathbf{u}_{\mathrm{h}}(t) \in \mathbb{R}^{m_{\mathrm{h}}}$. In general, the dimensions of the robot and human inputs may differ.

In the simplest case where $m = m_{\mathrm{h}} = m_{\mathrm{r}}$ with perfect kinematic alignment between the robot and the human, one would define $\mathbf{S}_{\mathrm{r}} = \mathbf{S}_{\mathrm{h}} = \mathbf{I}$ (identity matrix) and $\mathbf{u} = \mathbf{u}_{\mathrm{r}} + \mathbf{u}_{\mathrm{h}}$, as in the main text. In this case, the two rigid body dynamics are considered together, and the interaction torque is canceled. Similar matrices can be defined when focusing solely on the robot's perspective and attributing the interaction torque to the human input, as seen e.g. in.[1] This approach may be relevant when carrying a large load, such that the human dynamics can be neglected. However, the reshaping matrices $\mathbf{S}_{\mathrm{r}}$ and $\mathbf{S}_{\mathrm{h}}$ can be used for modeling more complex physical interaction considering joint misalignments and the intrinsic human dynamics.

Considering the matrix $\mathbf{B}(t) = \dfrac{\partial \mathbf{f}}{\partial \mathbf{u}}(\mathbf{x}_{\mathrm{d}}(t), \mathbf{u}_{\mathrm{d}}(t), t)$ from the linearization of the interaction dynamics $\dot{\mathbf{x}}(t) = \mathbf{f}(\mathbf{x}(t), \mathbf{u}(t), t)$ around a desired trajectory/control pair $[\mathbf{x}_{\mathrm{d}}(t), \mathbf{u}_{\mathrm{d}}(t)]$, and defining $\mathbf{B}_{\mathrm{r}}(t) \triangleq \mathbf{B}(t)\mathbf{S}_{\mathrm{r}}$ and $\mathbf{B}_{\mathrm{h}}(t) \triangleq \mathbf{B}(t)\mathbf{S}_{\mathrm{h}}$, we obtain the following local *affine human-robot interaction model*:

$$\dot{\boldsymbol{\xi}}(t) = \mathbf{A}(t)\boldsymbol{\xi}(t) + \mathbf{B}_{\mathrm{h}}(t)\mathbf{u}_{\mathrm{h}}(t) + \mathbf{B}_{\mathrm{r}}(t)\mathbf{u}_{\mathrm{r}}(t) + \mathbf{c}(t)\,. \tag{2}$$

In these settings, the Nash equilibrium can be obtained by solving the coupled Riccati-like differential

equations (see Corollary 6.5 in,[2] page 323):

$$\begin{aligned}
-\dot{\mathbf{P}}_{\mathrm{i}} &= \mathbf{F}^{\top}\mathbf{P}_{\mathrm{i}} + \mathbf{P}_{\mathrm{i}}\mathbf{F} + \mathbf{Q}_{\mathrm{i}} + \sum_{\mathrm{j}\in\{\mathrm{r},\mathrm{h}\}} \mathbf{P}_{\mathrm{j}}\mathbf{B}_{\mathrm{j}}\mathbf{R}_{\mathrm{j}}^{-1}\mathbf{R}_{\mathrm{ij}}\mathbf{R}_{\mathrm{j}}^{-1}\mathbf{B}_{\mathrm{j}}^{\top}\mathbf{P}_{\mathrm{j}}\,, \\
-\dot{\boldsymbol{\alpha}}_{\mathrm{i}} &= \mathbf{F}^{\top}\boldsymbol{\alpha}_{\mathrm{i}} + \mathbf{P}_{\mathrm{i}}\boldsymbol{\beta} + \sum_{\mathrm{j}\in\{\mathrm{r},\mathrm{h}\}} \mathbf{P}_{\mathrm{j}}\mathbf{B}_{\mathrm{j}}\mathbf{R}_{\mathrm{j}}^{-1}\mathbf{R}_{\mathrm{ij}}\mathbf{R}_{\mathrm{j}}^{-1}\mathbf{B}_{\mathrm{j}}^{\top}\boldsymbol{\alpha}_{\mathrm{j}}\,, \\
\mathbf{F} &\triangleq \mathbf{A} - \sum_{\mathrm{i}\in\{\mathrm{r},\mathrm{h}\}} \mathbf{B}_{\mathrm{i}}\mathbf{R}_{\mathrm{i}}^{-1}\mathbf{B}_{\mathrm{i}}^{\top}\mathbf{P}_{\mathrm{i}}\,, \quad \boldsymbol{\beta} \triangleq \mathbf{c} - \sum_{\mathrm{i}\in\{\mathrm{r},\mathrm{h}\}} \mathbf{B}_{\mathrm{i}}\mathbf{R}_{\mathrm{i}}^{-1}\mathbf{B}_{\mathrm{i}}^{\top}\boldsymbol{\alpha}_{\mathrm{i}}\,.
\end{aligned} \tag{3}$$

This complete solution includes $\mathbf{R}_{\mathrm{hr}}$, which could be used to model that the human seeks to minimize the robot effort (whereas in the main text we only assumed that the robot assists the human using $\mathbf{R}_{\mathrm{rh}}$). In the main text, we instead set $\mathbf{R}_{\mathrm{hr}} = \mathbf{0}$ and used the notation $\mathbf{R}_{\mathrm{hh}} = \mathbf{R}_{\mathrm{h}}$ and $\mathbf{R}_{rr} = \mathbf{R}_{\mathrm{r}}$. In this general affine-quadratic case, the Nash-optimal control laws are given by:

$$\mathbf{u}_{\mathrm{i}}(t, \boldsymbol{\xi}(t)) = -\mathbf{R}_{\mathrm{i}}^{-1}\mathbf{B}_{\mathrm{i}}(t)^{\top}[\boldsymbol{\alpha}_{\mathrm{i}}(t) + \mathbf{P}_{\mathrm{i}}(t)\boldsymbol{\xi}(t)]\,, \quad \mathrm{i}\in\{\mathrm{r},\mathrm{h}\}\,, \quad t \in [\tau, \tau + \triangle_{\mathrm{p}}]\,. \tag{4}$$

The MPG control method presented in the main text can readily be applied with these modified equations.

Supplementary Results

Here, we present supplementary results that show how close is MPG prediction to the actual human behavior. Additional analysis were conducted on data from experiment 2, where we calculated the root mean square error (RMSE) between the measured human torque $\tilde{u}_{\mathrm{h}}$ and the MPG predicted torque $\hat{u}_{\mathrm{h}}$ noted $\tilde{u}_{h\mathrm{RMSE}}$. Fig. 1A shows the error during the first 30 second of the trials where the average

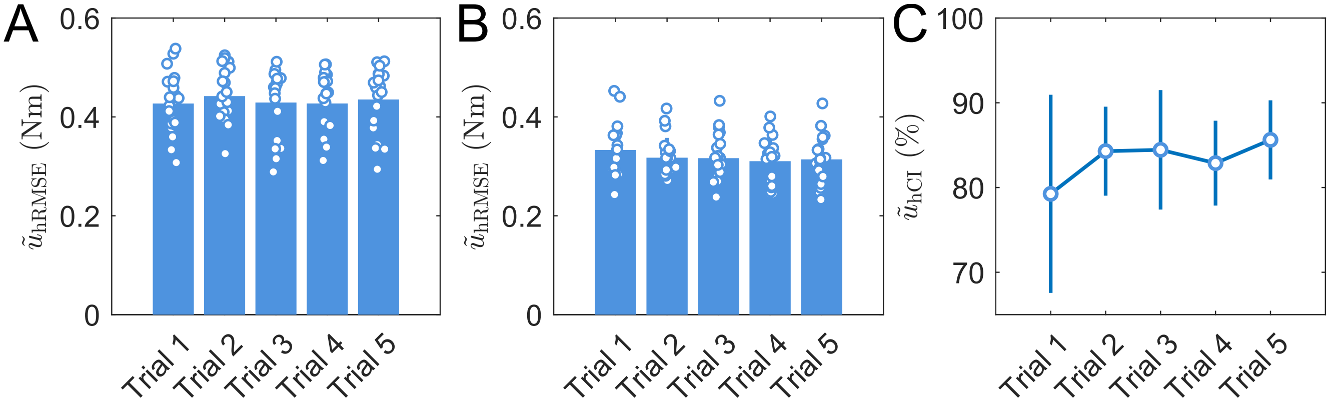

Figure 1. Measured participants torque vs MPG estimated torque. Results are displayed over consecutive MPG trials of experiment 2. **A.** Root mean square error of the MPG estimated participants applied torques $u_{h\mathrm{RMSE}}$ during the first quarter of the trials. **B.** Root mean square error of the MPG estimated participants applied torques $u_{h\mathrm{RMSE}}$ during the last quarter of the trials. **C.** Coordination index between the participants measured torques and the MPG torques during the last quarter of the trials.

error between all trials was $\bar{u}_{h\mathrm{RMSE}} = 0.43$ Nm. No significant difference was observed between the trials ($p = 0.68$). Fig. 1B shows the error during the last 30 second of the trials where the average error was $\bar{u}_{h\mathrm{RMSE}} = 0.31$ Nm. No significant difference was observed between the trials ($p = 0.22$).

However, the decrease of the error between the first and the last 30 seconds of the trials was statistically significant ($p < 0.001$, $r > 0.9$). This results suggest that the prediction improves as the estimation of the participants cost parameter reaches the Nash equilibrium plateau independently of the human adaptation across trials. Fig. 1C shows the evolution of the coordination index during the last 30 seconds between the measured participants toques and the MPG predicted torques across the trials. Where the coordination index significantly increased across trials ($p < 0.05$ Kendall' $W = 0.02$, $\chi^2_F = 17.08$, post-hoc test showed significance for trial 1 vs trial 3 and trial 5, with $p < 0.05$).

Supplementary Note 2

In this supplementary note, we analyze the main differences between a finite- and infinite-horizon DG in human-robot interaction to understand how the choice of horizon affects the game formulation, the resulting Nash equilibrium and the control performance. This analysis is conducted in a simple trajectory tracking simulation with wrist flexion/extension movements up and down, consistent with our experiments.

Preliminary considerations

In the standard infinite-horizon formulation, the state and control matrices must be time-invariant and the motor controls of the human and robot agents (u_h and u_r) are defined through deviations, This enables the application of the standard DG solution (see pp. 336–7 in[2]). This formulation implicitly assumes that the equilibrium torques are zero, i.e. there is no steady force to counteract. These assumptions hold in setups such as that of,[1] where interaction was constrained to a horizontal plane and the equilibrium was unaffected by gravity. In that context, the control represented pure feedback terms around a zero-torque equilibrium, and both the shared state and agents cost functions could be expressed as deviations from this equilibrium.

In contrast, our setup involves varying gravitational terms, so that even at equilibrium the system requires a nonzero steady torque. Modeling our scenario with an infinite-horizon approach therefore requires (i) linearizing the dynamics around a fixed point to obtain time-invariant matrices, and (ii) an *ad hoc* assumption about how the agents share this steady torque. This assumption is implemented through the parameter γ that operates similarly to λ used in the cost functions, but at the dynamics level, as described in the Interaction dynamics section.

Moreover, in such purely feedback formulations, when the task is well performed—i.e. when the tracking error $\boldsymbol{\xi} = \mathbf{0}$ and $\mu_\mathrm{h} = 0$—the human cost function becomes unobservable. This is explicitly acknowledged in[1] (at p. 38, by Eq. (10)): *"[...] the partners' controllers and cost functions converge to the correct values if $\boldsymbol{\xi}$ is persistently exciting"*. Hence, this previous formulation required task deviations to observe the human cost function, which is feasible in reaching tasks (as the error needs to vanish only at the end of the movement) but not in continuous tracking.

Our finite-horizon approach overcomes these limitations. It (i) can handle time-varying state and control matrices, naturally through the backward integration of the ODEs implementing the DG, and (ii) directly

optimizes u_h and u_r considering both feedback and the feedforward components that emerge from the task dynamics. As a first consequence, the parameter γ is not necessary, allowing the agents to autonomously determine how to share the efforts required to accomplish the task in real time, without *ad hoc* constraints. Second, the agents' control is not null even when $\boldsymbol{\xi} = \mathbf{0}$, enabling the human strategy to remain observable when the task is perfectly executed.

Below, we describe how these considerations impact the formulation of the problem, the tracking performance, and the contributions of the agents for different human behaviors.

Interaction dynamics

We consider the coupled human-robot-load system:

$$\mathbf{f}(\mathbf{x}, u, t) = \begin{bmatrix} \dot{q} \\ [u_\mathrm{h} + u_\mathrm{r} - \mathcal{G}(q) - \mathcal{D}\dot{q}] \,/\, \mathcal{I} \end{bmatrix}. \tag{5}$$

where q is the position, u_r and u_h are the robot and human applied torques respectively, $\mathcal{I}$, $\mathcal{D}$ and $\mathcal{G}$ are the inertia, damping and the (nonlinear) gravitational term, respectively. Linearization around some fixed desired trajectory-control pair $(\mathbf{x}_\mathrm{d}, u_\mathrm{d})$ for infinite-horizon setup yields:

$$\dot{\boldsymbol{\xi}}(t) = \mathbf{A}\boldsymbol{\xi}(t) + \mathbf{B}\left[\mu_\mathrm{r}(t) + \mu_\mathrm{h}(t)\right], \quad \boldsymbol{\xi} = \mathbf{x} - \mathbf{x}_\mathrm{d}, \quad \mathbf{x} = \begin{bmatrix} q \\ \dot{q} \end{bmatrix}, \quad \mathbf{x_d} = \begin{bmatrix} q_\mathrm{d} \\ \dot{q}_\mathrm{d} \end{bmatrix}, \tag{6}$$
$$\mathbf{A} \triangleq \frac{\partial \mathbf{f}}{\partial \mathbf{x}}\Big|_{[\mathbf{x}_\mathrm{d}(\tau+\triangle_\mathrm{p}),\, u_\mathrm{d}(\tau+\triangle_\mathrm{p})]}, \quad \mathbf{B} \triangleq \frac{\partial \mathbf{f}}{\partial u}\Big|_{[\mathbf{x}_\mathrm{d}(\tau+\triangle_\mathrm{p}),\, u_\mathrm{d}(\tau+\triangle_\mathrm{p})]}.$$

where τ is the actual time, $\triangle_\mathrm{p}$ is the planing horizon fixed in the finite-horizon approach, and the control inputs are defined as $\mu_\mathrm{r} = u_\mathrm{r} - \gamma u_\mathrm{d}$ and $\mu_\mathrm{h} = u_\mathrm{h} - (1-\gamma)u_\mathrm{d}$. As previously mentioned, the infinite-horizon imposes to linearize around a fixed point, which we define at $t = \tau + \triangle_\mathrm{p}$, where $\triangle_\mathrm{p} = 0.5\,\mathrm{s}$ is the planning horizon of the finite-horizon formulation. Hence, although we will apply DG on an infinite horizon, the target still corresponds to some upcoming state that the controls need to fulfill.

The formulation of the finite-horizon approach remains unchanged as presented in our method.

MPG infinite horizon controller implementation

The human and robot optimize their respective cost functions in the infinite horizon setup:

$$J_\mathrm{h} = \frac{1}{2}\int_\tau^\infty \boldsymbol{\xi}^\top \mathbf{Q}_\mathrm{h}\boldsymbol{\xi} + R_\mathrm{h}\mu_\mathrm{h}^2 \; dt\,, \tag{7}$$

$$J_\mathrm{r} = \frac{1}{2}\int_\tau^\infty \boldsymbol{\xi}^\top \mathbf{Q}_\mathrm{r}\boldsymbol{\xi} + R_\mathrm{r}\mu_\mathrm{r}^2 + R_\mathrm{rh}\mu_\mathrm{h}^2 \; dt\,. \tag{8}$$

For this comparison, we set $R_\mathrm{h} = 1$, $R_\mathrm{r} = 0.5 + (1-\lambda)$, and $R_\mathrm{rh} = 0.5 + \lambda$ similarly to our finite-horizon setup. Note that the cross term R_rh lacks physical meaning here, as minimizing μ_h does not correspond to a physically interpretable interaction variable. This highlights another limitation of the infinite-horizon framework: it cannot represent cooperative terms that correspond to physically meaningful shared effort.

The optimal control is obtained by solving the coupled Riccati-like equations (see p. 337, Eq. (6.87) in[2]) using Newton-Kleinman algorithm at each timestep, using the same MPG approach as for the finite horizon:

$$\begin{cases} \mathbf{F}^\top \mathbf{P}_\mathrm{h} + \mathbf{P}_\mathrm{h}\mathbf{F} + \mathbf{P}_\mathrm{h}\mathbf{B}R_\mathrm{h}^{-1}\mathbf{B}^\top \mathbf{P}_\mathrm{h} + \mathbf{Q}_\mathrm{h} = 0\,, \\ \mathbf{F}^\top \mathbf{P}_\mathrm{r} + \mathbf{P}_\mathrm{r}\mathbf{F} + \mathbf{P}_\mathrm{r}\mathbf{B}R_\mathrm{r}^{-1}\mathbf{B}^\top \mathbf{P}_\mathrm{r} + \mathbf{P}_\mathrm{h}\mathbf{B}R_\mathrm{h}^{-1}R_\mathrm{rh}R_\mathrm{h}^{-1}\mathbf{B}^\top \mathbf{P}_\mathrm{h} + \mathbf{Q}_\mathrm{r} = 0\,, \\ \mathbf{F} = \mathbf{A} - \mathbf{B}R_\mathrm{r}^{-1}\mathbf{B}^\top \mathbf{P}_\mathrm{r} - \mathbf{B}R_\mathrm{h}^{-1}\mathbf{B}^\top \mathbf{P}_\mathrm{h}\,, \end{cases} \tag{9}$$

and the optimal feedback commands are:

$$\mu_\mathrm{i}(\tau) = -R_\mathrm{r}^{-1}\mathbf{B}^\top \mathbf{P}_\mathrm{r}\boldsymbol{\xi}\,, \quad \mu_\mathrm{h}(\tau) = -R_\mathrm{h}^{-1}\mathbf{B}^\top \mathbf{P}_\mathrm{h}\boldsymbol{\xi}\,. \tag{10}$$

Simulation settings

We simulated the controllers with the following parameters: $\mathcal{I} = 0.1\,\mathrm{kg\,m^2}$, $\mathcal{D} = 0.1\,\mathrm{kg\,m^2\,s^{-1}}$, $m = 1\,\mathrm{kg}$, $l = 0.1\,\mathrm{m}$, and $g = 9.81\,\mathrm{m\,s^{-2}}$. The nominal control input was:

$$u_\mathrm{d}(t) = \mathcal{I}\,\ddot{q}_\mathrm{d}(t) + \mathcal{D}\,\dot{q}_\mathrm{d}(t) + mgl\sin[q_\mathrm{d}(t)]\,, \tag{11}$$

with the target trajectory defined as:

$$q_\mathrm{d}(t) = \frac{\pi}{4}\left[1 + \cos(\pi t)\right]\,, \tag{12}$$

the initial position and velocity were set at $q(0) = \pi/4$, $\dot{q}(0) = 0$, and the cost matrices were set as $\mathbf{Q}_\mathrm{r} = \mathbf{Q}_\mathrm{max}/2$ and $\mathbf{Q}_\mathrm{h} = \zeta\,\mathbf{Q}_\mathrm{max}$, $0 \leq \zeta \leq 1$, with $\mathbf{Q}_\mathrm{max} = \mathrm{diag}(200, 1)$.

Results

Tracking performance

Fig. 2 A,B illustrate how the finite- and infinite-horizon DG controllers track the reference trajectory (for the direct DG problem with known costs). The mean position and velocity errors shown in Panels C and D indicate that the infinite-horizon controller exhibits substantially higher errors than the finite-horizon controller (42.5% in position and 33.3% in velocity). This degradation in performance is mainly due to the linearization approximation, where at each time step the infinite-horizon approach assumes a fixed target at $t = \tau + \triangle_\mathrm{p}$ to yield time-invariant state matrices, whereas in the finite horizon approach the linearization is performed along the desired trajectory over the future finite horizon $t \in [\tau,\ \tau + \triangle_\mathrm{p}]$. These results highlight the importance of the finite-horizon formulation for accurately tracking both the desired trajectory and the dynamics.

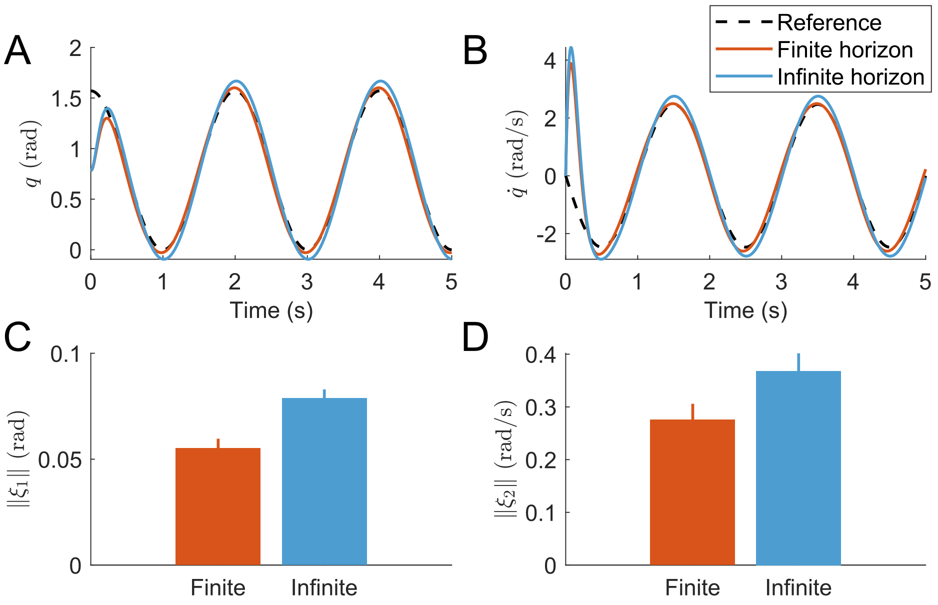

Figure 2. Tracking performance. A. Position evolution over time. **B.** Velocity evolution over time. **C.** Mean position error with standard error over 5 seconds of tracking for finite- and infinite-horizon DG controllers. **D.** Mean velocity error with standard error over 5 seconds of tracking for finite- and infinite-horizon DG controllers.

Effort sharing comparison

We further compared the effort sharing between the finite- and infinite-horizon formulations. Fig 3 shows the mean applied torque of the robot and human, during 5 seconds of the tracking task, for different values of the human cost matrix $\mathbf{Q}_\mathrm{h}$ and the parameter λ. We observe that varying either $\mathbf{Q}_\mathrm{h}$ or λ has a negligible effect in the effort sharing with the infinite-horizon approach, unlike in the finite-horizon approach. This difference arises because, in the infinite horizon case, the total applied effort consists of a shared feedback correction term and a fixed feedforward component. The latter compensates for the steady torque, which are predominant, resulting in a fixed effort distribution between agents when the task is well performed.

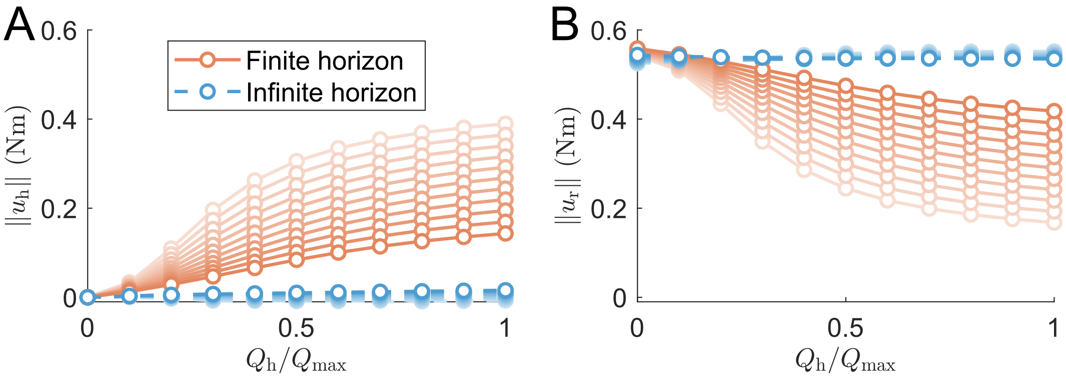

Figure 3. Mean human and robot effort over 5 s of tracking as a function of $\mathbf{Q}_\mathrm{h}/\mathbf{Q}_\mathrm{max}$ for different λ values and $\gamma = 1$.

Although varying γ can redistribute the effort between agents (Fig. 4), it rigidly dictates the effort-sharing

pattern. This means that the infinite-horizon formulation prevents any individualization or significant co-adaptation, since variations in $\mathbf{Q}_\mathrm{h}$ have a negligible impact when the task is well executed with $\boldsymbol{\xi} \simeq \mathbf{0}$. Thus, the infinite-horizon approach loses a principal advantage of the DG control formulation: its dynamic adaptability.

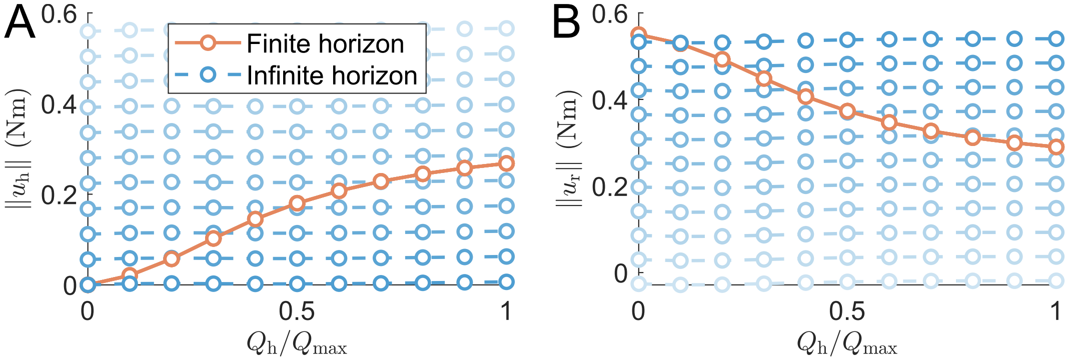

Figure 4. Mean human and robot effort over 5 seconds of tracking as a function of $\mathbf{Q}_\mathrm{h}/\mathbf{Q}_\mathrm{max}$ for different γ values, for $\lambda = 0.5$.

In contrast, the finite-horizon formulation incorporates the evolving task dynamics, allowing both $\mathbf{Q}_\mathrm{h}$ and λ to influence the resulting control effort. As shown in Fig. 3, this results in distinct variations in human and robot applied torques as these parameters change, enabling flexible and personalized effort sharing. Moreover, our experiments demonstrate that this mechanism allows the robot to shape the interaction relationship with the human user, an ability that could, for example, be used to optimize physical training protocols.

In conclusion, the finite-horizon is more flexible and exhibits unique properties to model the co-adaptation of two intelligent agents sharing effort in a load-carrying tracking task.

References

1. Y. Li, G. Carboni, F. Gonzalez, D. Campolo, and E. Burdet, "Differential game theory for versatile physical human–robot interaction," Nature Machine Intelligence, vol. 1, no. 11, p. 36–43, Jan. 2019.

2. T. Başar and G. J. Olsder, Dynamic Noncooperative Game Theory, 2nd Edition, ser. Classics in Applied Mathematics. Society for Industrial and Applied Mathematics, Jan. 1998. [Online]. Available: https://epubs.siam.org/doi/book/10.1137/1.9781611971132
